# Wrist-Based Accelerometers and Visual Analog Scales as Outcome Measures for Shoulder Activity During Daily Living in Patients With Rotator Cuff Tendinopathy: Instrument Validation Study

**DOI:** 10.2196/14468

**Published:** 2019-12-03

**Authors:** Samuel Larrivée, Frédéric Balg, Guillaume Léonard, Sonia Bédard, Michel Tousignant, Patrick Boissy

**Affiliations:** 1 Research Center on Aging Centre intégré universitaire de santé et de services sociaux de l'Estrie Centre Hospitalier Universitaire de Sherbrooke Sherbrooke, QC Canada; 2 Department of Surgery, Division of Orthopedics Faculty of Medicine and Health Sciences Université de Sherbrooke Sherbrooke, QC Canada; 3 Research Center of CHUS Centre intégré universitaire de santé et de services sociaux de l'Estrie Centre Hospitalier Universitaire de Sherbrooke Sherbrooke, QC Canada; 4 School of Rehabilitation Faculty of Medicine and Health Sciences Université de Sherbrooke Sherbrooke, QC Canada

**Keywords:** shoulder, wearable sensors, activity count, validation, test-retest, sensitivity to change

## Abstract

**Background:**

Shoulder pain secondary to rotator cuff tendinopathy affects a large proportion of patients in orthopedic surgery practices. Corticosteroid injections are a common intervention proposed for these patients. The clinical evaluation of a response to corticosteroid injections is usually based only on the patient’s self-evaluation of his function, activity, and pain by multiple questionnaires with varying metrological qualities. Objective measures of upper extremity functions are lacking, but wearable sensors are emerging as potential tools to assess upper extremity function and activity.

**Objective:**

This study aimed (1) to evaluate and compare test-retest reliability and sensitivity to change of known clinical assessments of shoulder function to wrist-based accelerometer measures and visual analog scales (VAS) of shoulder activity during daily living in patients with rotator cuff tendinopathy convergent validity and (2) to determine the acceptability and compliance of using wrist-based wearable sensors.

**Methods:**

A total of 38 patients affected by rotator cuff tendinopathy wore wrist accelerometers on the affected side for a total of 5 weeks. Western Ontario Rotator Cuff (WORC) index; Short version of the Disability of the Arm, Shoulder, and Hand questionnaire (QuickDASH); and clinical examination (range of motion and strength) were performed the week before the corticosteroid injections, the day of the corticosteroid injections, and 2 and 4 weeks after the corticosteroid injections. Daily Single Assessment Numeric Evaluation (SANE) and VAS were filled by participants to record shoulder pain and activity. Accelerometer data were processed to extract daily upper extremity activity in the form of active time; activity counts; and ratio of low-intensity activities, medium-intensity activities, and high-intensity activities.

**Results:**

Daily pain measured using VAS and SANE correlated well with the WORC and QuickDASH questionnaires (r=0.564-0.815) but not with accelerometry measures, amplitude, and strength. Daily activity measured with VAS had good correlation with active time (r=0.484, *P*=.02). All questionnaires had excellent test-retest reliability at 1 week before corticosteroid injections (intraclass correlation coefficient [ICC]=0.883-0.950). Acceptable reliability was observed with accelerometry (ICC=0.621-0.724), apart from low-intensity activities (ICC=0.104). Sensitivity to change was excellent at 2 and 4 weeks for all questionnaires (standardized response mean=1.039-2.094) except for activity VAS (standardized response mean=0.50). Accelerometry measures had low sensitivity to change at 2 weeks, but excellent sensitivity at 4 weeks (standardized response mean=0.803-1.032).

**Conclusions:**

Daily pain VAS and SANE had good correlation with the validated questionnaires, excellent reliability at 1 week, and excellent sensitivity to change at 2 and 4 weeks. Daily activity VAS and accelerometry-derived active time correlated well together. Activity VAS had excellent reliability, but moderate sensitivity to change. Accelerometry measures had moderate reliability and acceptable sensitivity to change at 4 weeks.

## Introduction

Shoulder pain is a frequent problem in adults of all ages [1]. A large proportion of shoulder pains is caused by rotator cuff tendinopathy [2,3], a chronic degenerative disease affecting rotator cuff tendons in the shoulder [4]. Patients with rotator cuff tendinopathy generally experience pain when performing activities of daily living [5]. Shoulder pain is often accompanied by stiffness and weakness that can degenerate into declining shoulder function, diminished work capabilities, and overall decreased quality of life [5,6]. Conservative treatment for rotator cuff tendinopathy usually starts with nonsteroidal anti-inflammatory medications (NSAID) and physical therapy to regain full range of motion and restore scapular control [7,8]. Corticosteroid injections (CSI) in the subacromial space are also used in conjunction with NSAIDs and physical therapy to further alleviate the pain symptoms by reducing the inflammatory response, and facilitate mobilization of the shoulder. As a last resort, a surgical option such as bursectomy or acromioplasty can be offered to patients with persistent rotator cuff tendinopathies [9]. Currently, evaluation of the response to treatment is based on patient-reported outcomes using subjective measures, such as questionnaires and pain scores [7], which do not necessarily correlate with real-life function of the shoulder and do not capture efforts made by the patients in mobilizing their shoulder during daily activities.

Up to 40 different questionnaires have been proposed as outcome measures for the follow-up of shoulder pathologies [10]. The Disability of the Arm Shoulder and Hand (DASH), its short version (QuickDASH), the Shoulder Pain and Disability Index, the American Shoulder and Elbow Society score, and the Constant-Murley scores have been well validated, but none are consistently recommended in the literature [11]. Some questionnaires, such as the Western Ontario Rotator Cuff Index (WORC), have also been developed to assess patients with rotator cuff tendinopathy specifically [12]. However, all these activity and function questionnaires assess perceived capacity and activity, which have different bias inherent to this type of evaluation. Other simpler measures such as pain or activity (measured with visual analog scales [VAS]) and the Single Assessment Numeric Evaluation (SANE) have, however, rarely been studied in the context of patients with rotator cuff tendinopathy. Objective clinical examinations such as strength and range of motion are rarely correlated with patients’ subjective assessment of function and have usually poor sensitivity to change in the context of rotator cuff tendinopathy [13-15].

Objective outcome measures based on wearable motion sensors could prove useful in the clinical evaluation of real-life shoulder activity and mobilization of patients with rotator cuff tendinopathy as well as an outcome to measure the effect of different treatments on shoulder activity and function. Multiple authors have used raw sensor data from inertial sensors (accelerometers and gyroscope) or orientation data from Attitude and Heading Reference Systems positioned on different segments to capture shoulder activity. Numerous techniques and algorithms have been proposed, such as the range of angular velocities and linear accelerations measured during a set of standardized tasks [16-18], detection of active time [19,20], measurement of shoulder elevation angles in clinic or daily life [21-28], and movement classification algorithms [29,30]. Most of these methods are not suitable for continuous monitoring over long periods, as they require either many devices on the same arm or one on each limb, or that the device be positioned at the humerus, all of which affect long-term adherence of wearing the devices by the participants. Consequently, the data collection for all the methods presented above was limited to short periods of 8 hours at most or to standardized evaluation in the clinic, which is not a valid representation of the patient’s real-life activities.

Activity counts (ACs), a manufacturer-specified unit obtained from raw accelerometer output [31,32], could prove useful as a way to quantify upper extremity use during a longer follow-up period. ACs were initially used to quantify whole-body physical activity using accelerometers worn at the waist or wrist [33], but have been since adapted to assess upper extremity impairments associated with different pathologies and to monitor the impact of rehabilitation. They can be obtained in three broad ways: (1) counting how many times the raw accelerometer data cross a predetermined threshold, (2) a rolling window method where the count is determined as the highest acceleration in that window, or (3) calculating the area under the curve of the acceleration signal for each window [31,32]. Acuna et al [34] measured ACs derived from humerus-worn and wrist-worn accelerometers and observed a good correlation between both approaches. This could justify the wrist positioning as a valid position to quantify shoulder activity, which can improve participant acceptability of a continuous monitoring protocol in their own environment [35]. Lawinger et al [36] used wrist-based accelerometers to analyze different shoulder rehabilitation exercises and activities of daily living involving the upper extremity in a clinical setting. They demonstrated that ACs were sensitive enough to detect low-velocity exercises and that a good correlation could be found between the amount of movement performed and the measured AC (r=0.93, *P*<.001). To our knowledge, however, ACs have not been validated in the setting of patients with rotator cuff tendinopathy and their convergent validity, fidelity, and sensitivity to change are not known in this population.

Hence, the aims of this study were (1) to evaluate and compare convergent validity, test-retest reliability, and sensitivity to change of known clinical assessments of shoulder function to wrist-based accelerometer measures of shoulder activity during daily living in patients with rotator cuff tendinopathy and (2) to determine the acceptability and compliance of using wrist-based wearable sensors, which is an outcome measure to study the effects of CSI on rotator cuff tendinopathy.

## Methods

### Participants

Patients with unilateral or bilateral rotator cuff tendinopathy who were candidates for receiving a CSI were recruited from the orthopedic clinic of the Sherbrooke University Hospital Centre (Centre Hospitalier Universitaire de Sherbrooke [CHUS] - CIUSS de l’Estrie) and by referral from local physiotherapists and general practitioners. Rotator cuff tendinopathy was confirmed by a clinical diagnosis based on examination (presence of a painful arc of movement and positive impingement tests) and symptom duration of at least 9 months. The patients were screened by a medical student, and the diagnosis was confirmed by a fellowship-trained shoulder surgeon. Participants were excluded if they presented any other painful pathology of the shoulder (shoulder osteoarthritis, capsulitis, cervical pain radiating to the shoulder, rheumatic disease, etc), had a history of fracture or surgery on the affected shoulder, or received a shoulder CSI in the last 3 months. Significant rotator cuff tears were excluded by physical examination and diagnostic imaging, if available. The Ethics Review Board of the CIUSSS de l’Estrie-CHUS approved the protocol, and informed consent was obtained from each participant prior to his/her inclusion in the study.

### Study Design and Assessments

This is an embedded methodological study within a pilot randomized controlled trial on the effect of CSI and the addition of a sham or real treatment of transcranial direct current stimulation (tDCS) on shoulder function and activity in patients with rotator cuff tendinopathy. tDCS is an experimental treatment currently being investigated for chronic pain [37]. As part of this main study, participants were randomized 2 weeks after receiving a CSI to additionally receive tDCS treatment, placebo tDCS, or no intervention. Participants were followed up for a total of 5 weeks after CSI with assessments on patient-reported outcomes from questionnaires and clinical examinations at 0-, 1-, 3- and 5-week endpoints.

For this study, the participants were asked to wear an accelerometer logger called the WIMU-GPS (Wireless Inertial Measurement Unit with GPS) on the wrist of the affected shoulder for the whole duration of the study, from morning to bedtime. Participants were asked to answer daily questionnaires on shoulder pain levels and the relative usage of their upper extremity in the form of two VAS. At the 1-week evaluation endpoint, participants received a CSI in the affected shoulder. The CSI (1 ml of 40 mg/mL methylprednisolone and 4 mL of 1% [10 mg/mL] xylocaine injected using a 25-bore 1.5-inch needle) was performed in the subacromial space using the posterior approach by the same fellowship-trained orthopedic surgeon for all participants. Finally, at the 5-week evaluation endpoint, participants completed a short questionnaire about satisfaction and adherence to wearing the WIMU-GPS.

### Outcome Variables and Measures

#### Upper Limb Activity Measured Using Wrist-Based Accelerometry

**Figure 1:**
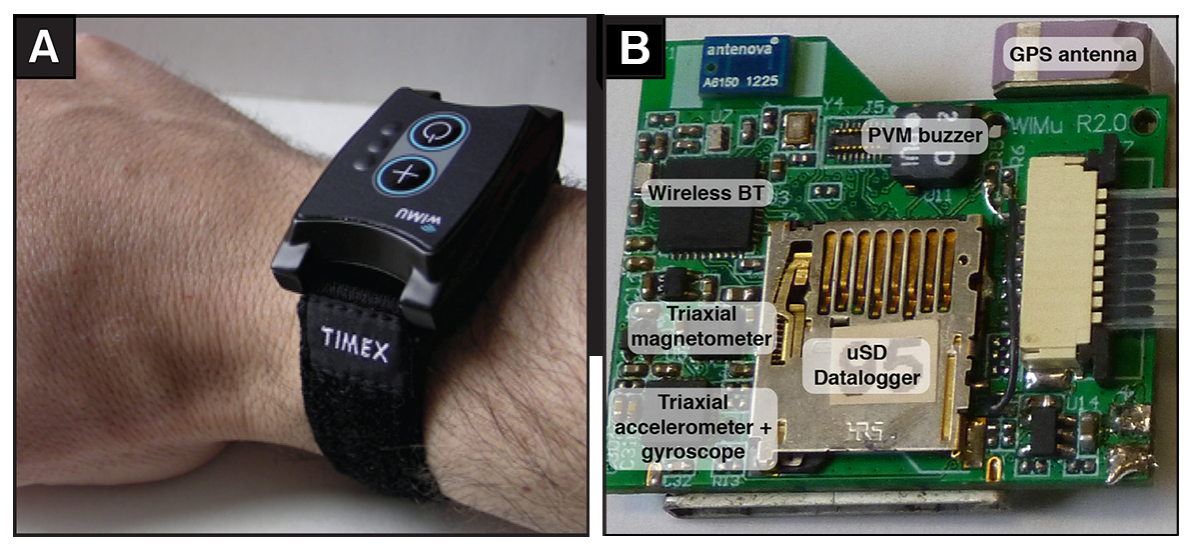
Overview of the WIMU-GPS (WIMU-GPS [Wireless Inertial Measurement Unit with GPS]) platform: (A) device worn on the wrist, and (B) printed circuit board and components. PVM: Pulse Variable Modulator buzzer chip; BT: Bluetooth chip; uSD: Micro-USD chip.

Upper limb activity was measured using 3D accelerometers embedded in a wearable activity monitoring system worn on the wrist. The WIMU-GPS [38] (Figure 1) is an activity-monitoring system developed at the Research Centre on Aging of the CIUSS de l’Estrie – CHUS to be used as a multisensor data-logging device with a small form factor to capture mobility and activity of individuals in their home environment over long-term recording periods. The third generation of the device currently consists of a triaxial accelerometer (2/4/8/16 g), a triaxial gyroscope (250/500/1000/2000 degrees/s), a triaxial magnetometer (0.8 Ga to 8.1 Ga), all sampled at 50 Hz, and a GPS (SiRFstarIV, 48 Channels) sampled at 1Hz. The data stream is then stored on an 8 GB microSD memory card. By using a 400 mAh Li-ion battery, the WIMU-GPS is able to record data continuously over a period of 10-14 hours on a full charge. The activated device was provided to participants at the beginning of the project. They were instructed to wear it on the wrist on the same side of their affected shoulder for all waking hours and to charge it at night. They were instructed to take it off during water-based activities. No instructions were given on how to turn on or off the device, as recordings are automatically paused when the battery is low or charging and are set to restart once it is disconnected from the power supply. At each endpoint, data were downloaded from the microSD chip onto a portable computer and the WIMU-GPS was reset. Data from each visit day were deleted, as these days would have been incomplete and the data would have possibly been invalidated by observation bias and patients disturbing their usual routine to attend the appointments.

**Figure 2:**
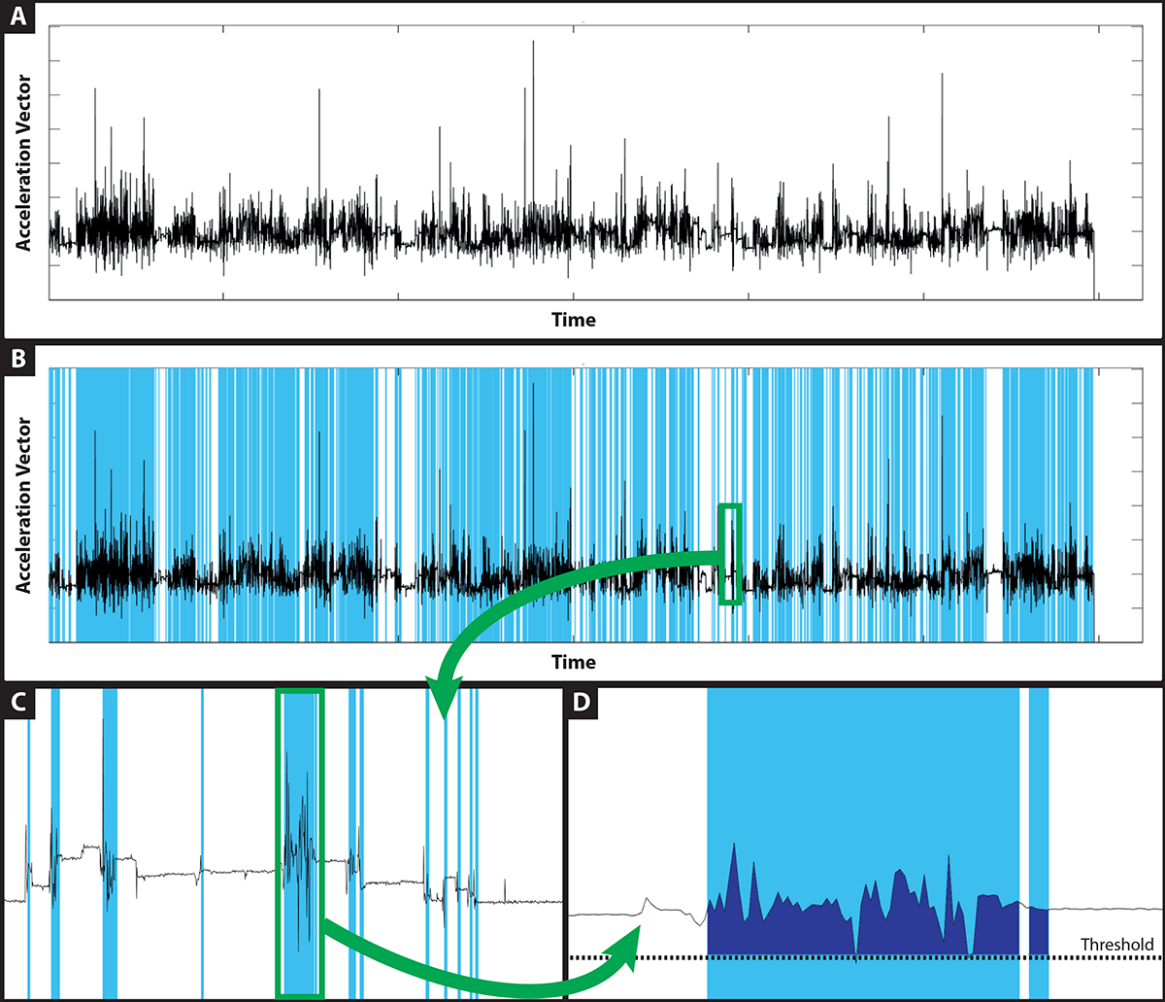
Processing of accelerometer data into active time, activity count, low-intensity activity, medium-intensity activity, and high-intensity activity. (A) Combined acceleration vector. (B, C) Periods of active time are identified in areas where 50% of the data values over 10 seconds rolling windows are over a fixed threshold of 0.015 g. (D) Acceleration vector in active periods is integrated to produce activity count. A distribution of the activity count of all activities detected in the sample was created and with activities count below the 33rd percentile (activity count 90.0) was classified as low-intensity activity; activities between the 33rd and 66th percentile, as medium-intensity activity; and activities above the 66th percentile (activity count 180.0), as high-intensity activity.

For this study, raw data from the three accelerometers were first low-pass filtered (Butterworth, 1 Hz, 2nd order) to remove sensor noise, full-wave rectified and high-pass filtered (Butterworth, 5 Hz, 2nd Order) to remove the gravitational acceleration vector, and combined into a unique vector using square root sums [39]. Periods of “active time” in the recordings were identified in areas of the vector where 50% of the data values over a 10-second window were over a fixed threshold (0.015 g) [40]. ACs were then computed using the integration method (Figure 2). Four variables are derived from this AC: *active time,* reported as a ratio with total recorded time; mean *AC* per minute of active time, the proportion of *low-intensity* (LIA) and *medium-intensity* (MIA), and *high-intensity* activities (HIA). Activities with an AC below the 33rd percentile of all activities recorded during the project were classified as LIA, while the 33rd-66th percentiles were defined as MIA, ACs above the 66th percentile were defined as HIA. The two thresholds separating these three activity levels are an AC of 90.0 and 180.0 (for LIA-MIA and MIA-HIA, respectively). To obtain a better representation of the participants’ upper extremity daily usage and to allow easier comparison with other outcome measures, data are reported as a mean for each week of follow-up (preinjection week and second and fourth week postinjection). Weeks with less than 3 days of valid data were eliminated from the analysis.

#### Daily Activity and Pain Measured Using Visual Analog Scale and Single Assessment Numeric Evaluation

Participants were also given daily questionnaires to complete at home every day for the duration of the study. The short questionnaire included a 100-mm VAS evaluating their perceived level of pain in the last 24 hours (VAS_pain_), and another 100-mm VAS for the perceived level of upper extremity use in the last 24 hours (VAS_activity_). The SANE is a new short questionnaire that simply asks, “How would you rate your shoulder today as a percentage of normal (from 0 to 100% being normal)?” [41]. It has not been previously validated for patients with rotator cuff tendinopathy, but shows good convergent validity with Rowe and American Shoulder and Elbow Society scores (r=0.72 and 0.66, respectively) in a young population with shoulder instability [41]. Scores for the daily questionnaires are reported for three items as an arithmetic mean for each follow-up week as per the accelerometry measures (Multimedia Appendix 1).

#### Perceived Shoulder Function and Quality of Life

The WORC and the QuickDASH are two health-related quality of life questionnaires, which were completed by participants at each of the four visits. Both questionnaires have been validated for follow-up of rotator cuff tendinopathy and upper extremity pathologies and show excellent test-retest reliability and sensitivity to change [12,42-46]. The scores are reported on a scale of 0 to 100. On the WORC, a higher score indicates better quality of life, while it is the opposite for the QuickDASH.

#### Physical Measures

Shoulder strength and amplitude were measured at each visit by either one of two standardized evaluators: a medical student or physical therapist. A hand-held dynamometer was used to measure shoulder strength in movements preferentially involving the three major rotator cuff muscles: scapular plane elevation with thumb pointed down (Jobe maneuver) for supraspinatus muscle, external rotation at the side for infraspinatus, and internal rotation at the side for subscapularis [13]. An inclinometer was used to measure active shoulder range of motion amplitude in all planes of shoulder movement: abduction, flexion, scapular plane elevation, external rotation with shoulder at 90° of abduction, external rotation with arm at the side, and internal rotation with shoulder at 90° of abduction [47]. Internal rotation was also evaluated using the maximal vertebral level reached by the thumb with the hand at the back [47].

#### Global Rating of Change

The *Global Rating of Change Scale* (GRCS) is a 15-point scale that asks participants to rate their global perceived improvement ranging from “*a very great deal worse*” (–7) to “*a very great deal better*” (+7). Change from +4 (“moderately better”) to +7 is considered moderate to important change [48]. The GRCS was handed to participants at the 3- and 5-week endpoints.

#### Compliance, Reliability of Data, and Acceptability of Wrist-Worn Accelerometry

A short questionnaire at the end of the study asked participants to estimate the number of times they forgot to wear or charge the WIMU-GPS. Discomfort and disturbance secondary to wearing the device was measured using the 100-mm VAS, and free space was given for any additional comment (Multimedia Appendix 2). Log data from the WIMU-GPS were also used to determine how many days the accelerometer was not charged or malfunctioning. Compliance and reliability are reported as the percentage of missing days over the total number of participant-days of the study.

### Statistical Analyses

All statistical analyses were performed using SPSS statistics (v24.0 for Windows, IBM Corporation, Armonk, New York). An α value of 0.05 was used as the threshold for statistical significance. Normal distribution of variables was tested using a Shapiro-Wilk test. Currently, there is no gold standard to measure shoulder function in the setting of rotator cuff tendinopathy or shoulder pathologies [10]. Therefore, *convergent validity* was assessed by correlating accelerometry variables from the first week with scores from questionnaires and physical tests at the first evaluation (before the CSI). Normally distributed variables were correlated using a Pearson test, and Spearman correlations were used in the other case. *Test-retest reliability* of questionnaires and accelerometry and *intrajudge reliability* of clinical measures were computed using an intraclass correlation coefficient (ICC) between assessment done a week prior to the injection and the one done immediately before the injection. For daily measures, the mean of the three days following the first assessment was correlated to the mean of the 3 next days. To determine *sensitivity to change* of the instrument, we used a method to discriminate between participants with meaningful improvement and those who showed unchanged results [49]. Hence, participants were dichotomized into two groups using the GRCS at the 3- and 5-week evaluations as either perceiving a moderate to important change (improved group, GRCS≥4) or perceiving a mild change or less (stable group, GRCS>–4 and <4). This limit was used by Mintken et al [45] to determine clinically important change for shoulder questionnaires [45]. Standardized response means (SRM) [50] were calculated for both groups to compare sensitivity to change for each outcome variable. An SRM<0.2 is considered a minimal effect, while one between 0.2 and 0.49 is small, between 0.5 and 0.79 is moderate, and ≥0.8 is large [50]. Sensitive and specific instruments for change should show good SRM for the improved group and an SRM approaching zero for the stable group [51].

## Results

### Patients’ Characteristics

Thirty-eight participants aged 25-65 years (mean 48.8 years, SD 10.4 years) were included in the study. Sociodemographics and baseline scores on patient-reported outcome of shoulder function, pain, and clinical exam results before CSI are shown in Table 1. All participants received the CSI and attended the preinjection, intervention, and 4-week follow-up visits. One participant was unable to be present at the 2-week visit and was instead asked to send in completed questionnaires by mail.

**Table 1:** Participant characteristics and scores at the first visit.

Parameter		Value
Number of participants		38
**Sex, n (%)**		
	Female	18 (47.4)
	Male	20 (52.6)
Age (years), mean (SD)		48.8 (10.4)
Body mass index (kg/m^2^), mean (SD)		27.9 (5.0)
Smokers, n (%)		5 (13.2)
**Dominant hand, n (%)**		
	Right	32 (84.3)
	Left	6 (15.7)
**Affected shoulder, n (%)**		
	Dominant	20 (52.6)
	Nondominant	18 (47.4)
Time since onset of symptoms (months), mean (SD)		71.4 (79.3)
**Questionnaire scores (out of 100), mean (SD)**		
	WORC^a^	46.88 (18.86)
	QuickDASH	42.85 (18.07)
	Pain VAS^b,c^	54.94 (18.76)
	Activity VAS^c^	58.24 (20.82)
	SANE^d^	53.42 (17.92)
**Strength (kg), mean (SD)**		
	Jobe	8.11 (3.45)
	External rotation	9.27 (3.69)
	Internal rotation	13.60 (5.34)
**Range of motion (°), mean (SD)**		
	Abduction	161.86 (22.30)
	Flexion	159.49 (18.78)
	Scaption	163.75 (17.17)
	Internal rotation 90°	73.68 (14.22)
	Spinal level	8.68 (3.40)
	External rotation 90°	76.68 (14.22)
	External rotation 0°	61.57 (17.15)

^a^WORC: Western Ontario Rotator Cuff.

^b^VAS: Visual Analog Scale.

^c^Arithmetic mean for the first week.

^d^SANE: Single Assessment Numeric Evaluation – arithmetic mean for the first week.

### Convergent Validity

Although all questionnaires and clinical exams were completed at the first visit, only 24/38 accelerometers had valid data in the first week. The Shapiro-Wilk test confirmed normal distribution of all the data, and a Pearson test was used to calculate convergent validity between the different variables (Table 2). As expected, WORC and QuickDASH were well correlated (r=0.821, *P*<.001, data not shown in table). Both questionnaires had a moderate-to-strong correlation with the pain VAS (r=–0.815 and 0.637, respectively) and SANE (r=0.613 and –0.564, respectively), but showed no significant correlation with the activity VAS. None of the four accelerometry variables (AT, AC, LIA, MIA, or HIA) showed any significant correlation with the WORC, QuickDASH, pain VAS, SANE, or clinical measures. However, there was a moderate correlation between the activity VAS and AT (r=0.484, *P*=.02). This correlation was significative at *P*<.05, but this statistical significance was lost following Bonferroni correction for multiple comparisons. Generally, acceleration measures correlate well with each other.

**Table 2:** Convergent validity.

		Pain VAS^a,b^	Activity VAS^b^	SANE^b,c^	Active time^b^	Activity count ^b^	LIA^b^^,d^	MIA^b,e^	HIA^b,f^
**WORC^g^ (n=38)**		–0.815^h^	–0.062	0.613^h^	0.327	0.353	–0.235	–0.199	0.229
	*P* value	<.001	.71	<.001	.12	.09	.27	.35	.28
**QuickDASH^i^ (n=38)**		0.637^h^	0.177	–0.564^h^	0.024	-0.170	–0.033	–0.023	0.015
	*P* value	<.001	.30	<.001	.92	.45	.88	.92	.95
**Jobe strength (kg) (n=38)**		–0.303	-0.173	0.271	0.155	0.065	0.062	0.272	–0.237
	*P* value	.06	.30	.10	.47	.76	.77	.20	.26
**Abduction range of motion (°) (n=38)**		–0.175	0.326^j^	0.210	0.386	0.317	–0.251	–0.262	0.275
	*P* value	.29	.05	.21	.06	.13	.24	.22	.19
**Pain VAS (n=38)**		—^k^	0.326^j^	–0.583^h^	–0.128	-0.327	0.170	0.033	–0.071
	*P* value		.05	<.001	.55	.12	.43	.88	.74
**Activity VAS (n=38)**		—	—	–0.110	0.484^j^	0.195	–0.369	–0.341	0.364
	*P* value			.51	.02	.36	.08	.10	.08
**SANE (n=38)**		—	—	—	–0.013	–0.020	0.072	0.302	–0.262
	*P* value				.95	.93	.74	.15	.22
**Active time^b^ (n=24)**		—	—	—	—	0.469^j^	–0.761^h^	–0.439^j^	0.529^h^
	*P* value					.02	<.001	.03	.01
**Activity count^b^ (n=24)**		—	—	—	—	—	–0.621^h^	–0.674^h^	0.699^h^
	*P* value						<.001	<.001	<.001
**LIA^b^ (n=24)**		—	—	—	—	—	—	0.676^h^	–0.772^h^
	*P* value							<.001	<.001
**MIA^b^ (n=24)**		—	—	—	—	—	—	—	–0.990^h^
	*P* value								<.001

^a^VAS: Visual Analog Scale.

^b^These values are presented as the arithmetic mean for the first week.

^c^SANE: Single Assessment Numeric Evaluation.

^d^LIA: low-intensity activity.

^e^MIA: medium-intensity activity.

^f^HIA: high-intensity activity.

^g^WORC: Western Ontario Rotator Cuff.

^h^Significant correlations with Bonferroni correction at *P*<.008

^i^QuickDASH: short version of the Disability of the Arm, Shoulder, and Hand questionnaire.

^j^Significant correlations at *P*<.05.

^k^Not applicable.

### Test-Retest and Intrajudge Validity

One-week test-retest and intrajudge reliability for all of the outcomes measured are presented in Table 3. All participants were present for the second visit (intervention visit) and completed the questionnaires; however, 11 did not receive standardized range of motions and strength testing on that date. ICCs were used to derive the fidelity of all outcome measures, except for internal rotation measured from the spinal level (not a continuous variable), for which we used a weighted Kappa coefficient. There was enough data in 24 WIMU-GPS to proceed. All questionnaires had excellent reliability (ICC=0.883-0.950), and clinical measures had good to excellent reliability (ICC=0.601-0.960). Accelerometry measures such as AT (ICC=0.724), AC (ICC=0.621), MIA (ICC=0.674), and HIA (ICC=0.661) had good reliability. However, MIA (ICC=0.104) showed very weak reliability in comparison.

**Table 3:** Test-retest reliability. Values provided are intraclass correlation coefficient unless indicated otherwise.

Questionnaires (n=38)		Reliability
WORC^a^		0.902
QuickDASH^b^		0.883
Pain VAS^c^		0.924
Activity VAS		0.908
SANE^d^		0.950
**Strength (n=27)**		
	Jobe (kg)	0.770
	External rotation (kg)	0.960
	Internal rotation (kg)	0.952
**Range of motion (n=27)**		
	Abduction (°)	0.812
	Flexion (°)	0.932
	Scaption (°)	0.886
	Internal rotation 90° (°)	0.786
	External rotation (spinal level)	0.93^e^
	External rotation 90° (°)	0.601
	External rotation 0° (°)	0.845
**Acceleration data (n=24)**		
	Active time	0.724
	Activity count	0.621
	LIA^f^	0.104
	MIA^g^	0.674
	HIA^h^	0.661

^a^WORC: Western Ontario Rotator Cuff.

^b^QuickDASH: short version of the Disability of the Arm, Shoulder, and Hand questionnaire.

^c^VAS: Visual Analog Scale.

^d^SANE: Single Assessment Numeric Evaluation.

^e^Weighted Kappa coefficient.

^f^LIA: low-intensity activity.

^g^MIA: medium-intensity activity.

^h^HIA: high-intensity activity.

### Sensitivity to Change

#### Global Rating of Change Scale

GRCS was completed by all participants at the 2- and 4-week evaluations. At 2 weeks, 31 patients (81.6%) felt improvements, six (15.8%) felt no change, and only one (2.6%) deteriorated following the injection. In addition, 25 of the 31 improved subjects (73.7%) classified their improvement as large on the GRCS scale (from “A good deal better” to “A very great deal better”). At 4 weeks, the number of participants who still described an improvement dropped to 26 (68.4%), while 11 did not feel better than the preinjection phase (28.9%). Only one participant (2.6%) felt worse at the 4-week evaluation (the same participant at the 2-week evaluation).

#### Sensitivity to Change at 2 Weeks and 4 Weeks

Sensitivity to change at 2 weeks and 4 weeks for all the outcomes measured are presented in Table 4. All patients filled the questionnaires at 2 weeks, but one could not attend the physical examination. Seventeen accelerometers contained enough data at the preinjection week and at the second week postinjection to allow for analysis. Of the participants who described a large improvement on the GRCS, the WORC, QuickDASH, pain VAS, and SANE all showed a very strong effect (SRM=1.384-1.508). A moderate effect was also seen for activity VAS (SRM=0.568) and clinical measures. In contrast, only a small effect was seen with all accelerometry measures (SRM=0.017-0.246). Patients who described small or absent improvement generally showed a small effect on questionnaires (SRM=0.108-0.621) and clinical measures (SRM=0.010-0.419) with the exception of a large effect at the Jobe strength testing (SRM=1.067). Activity measures had a variable range of specificity to change.

Questionnaires and clinical examinations were completed for all patients at 4 weeks. Enough valid data were available in 13 accelerometers. AC, LIA, MIA, and HIA showed a large SRM (0.802-1.032) for participants who felt a significant improvement on the GRCS. However, AT only had a weak effect (SRM=0.064). For patients with a slight to nonexistent improvement on the GRCS, all accelerometer variables showed a weak effect (SRM=0.010-0.176). In a similar fashion to the 2-week data, WORC, QuickDASH, and SANE questionnaires, all showed a strong effect (SRM=1.039-2.094) on improved patients and a small to moderate effect on others. The activity VAS showed a moderate effect (SRM=0.507) on improved participants and a very small effect on participants without improvement. Clinical measures had very variable sensitivity and specificity to change at 4 weeks.

**Table 4:** Sensitivity to change at 2 and 4 weeks.

Outcome measure		Standardized response means from 0 to 2 weeks		Standardized response means from 0 to 4 weeks	
		Large improvement (n=25)	Slight or no improvement (n=13)	Large improvement (n=20)	Slight or no improvement (n=18)
WORC^a^		1.412	0.108	1.039	0.544
QuickDASH^b^		1.384	0.138	1.245	0.056
Pain VAS^c,d^		1.508	0.610	2.094	0.788
Activity VAS^d^		0.568	0.228	0.507	0.012
SANE^d,e^		1.395	0.621	1.712	1.214
**Strength (kg)**					
	Jobe	0.101	1.067	0.057	0.553
	External rotation	0.473	0.228	0.866	0.474
	Internal rotation	0.594	0.168	0.674	0.357
**Range of motion** **(°)**					
	Abduction	0.230	0.010	0.349	0.191
	Flexion	0.456	0.087	0.510	0.048
	Scaption	0.119	0.172	0.093	0.419
	Internal rotation 90°	0.420	0.081	0.740	0.136
	External rotation 90°	0.240	0.419	0.016	0.010
	External rotation 0°	0.251	0.097	0.040	0.008
**Accelerometry^d^**					
	Active time	0.082	0.103	0.064	0.176
	Activity count	0.246	1.050	0.888	0.010
	LIA	0.068	0.091	0.885	0.087
	MIA	0.026	0.733	0.802	0.012
	HIA	0.017	0.767	1.032	0.019

^a^WORC: Western Ontario Rotator Cuff.

^b^QuickDASH: short version of the Disability of the Arm, Shoulder, and Hand questionnaire.

^c^VAS: Visual Analog Scale.

^d^Calculated from the arithmetic mean of the pre-injection week, second week post, and fourth week post.

^e^SANE: Single Assessment Numeric Evaluation.

### Patient Compliance and Data Loss

Recording days had a mean of 9 hours 59 minutes (SD 2 hours 44 minutes) of data on the accelerometers. Participants reported having forgotten to wear the device for a total of 6.2% of the recording days and forgotten to charge it 2.0% of these days. In comparison, WIMU-GPS data log show that participants forgot to charge or wear the device on 7.4% of the recording days. A software malfunction unfortunately caused a data loss for 31.2% of the recording days, increasing the total loss of recording days to 38.6%. As such, 57.0% of the follow-up weeks’ accelerometry data were valid as per our predefined criteria. At the end of the study, participants reported minimal discomfort while wearing the device (mean 20.58 mm on a 100-mm VAS, SD 19.16 mm) and minimal inconvenience (mean 21.69 mm on a 100-mm VAS, SD 20.41 mm). Four participants voiced that the device tended to catch with their clothes, and three participants would have preferred it to be smaller and more discreet.

## Discussion

The primary objective of this study was to validate wrist-based accelerometer measures and VAS of shoulder activity during daily living in comparison to other known measures for patients with rotator cuff tendinopathy. Daily pain VAS and SANE showed good convergent validity compared to previously validated questionnaires, while activity VAS and accelerometer data did not. However, activity VAS and AT correlated well together prior to correction for multiple comparison. Reliability was excellent for pain and activity VAS and SANE, but moderate for accelerometry measures. Sensitivity to change was excellent for pain VAS and SANE at 2 and 4 weeks and moderate for activity VAS and accelerometry measures at 4 weeks only. Evaluating the acceptability and compliance to wrist-based sensors was a secondary objective, and the accelerometers were shown to be easily accepted by patients who reported high adherence to wear.

The convergent validity of already validated questionnaires (WORC, QuickDASH, pain VAS, and SANE) was excellent and alike what has been already reported [12,14,52,53]. There was no significant correlation between questionnaires and range of motion, but there was a significant correlation between WORC and external and internal rotation strength and between SANE and strength in the Jobe test. Since the pathology mostly affects the tendon [4], but not the other structures in the shoulder, it would be logical that strength had some correlation with reported function, while range of motion did not. Although activity VAS was correlated with AT, reported and recorded shoulder activity did not correlate with any questionnaire or clinical measures. This is in contrast with correlations shown between accelerometry measures and DASH, Simple Shoulder Test, and pain VAS obtained by Jolles et al [18] and Korver et al [54]. These were obtained using multiple accelerometers and a standardized protocol of movements in a clinical setting, differing significantly from our protocol of unrestricted home usage. In both studies, patients were affected by a mix of pathologies (rotator cuff tendinopathy, shoulder osteoarthritis, etc), and a control group was used. Upper extremity activity might also represent a much different construct than that tested in questionnaires such as the WORC and DASH, and pain and subjective function are not necessarily linked with activity and use of the upper extremity. The correlation between AT and activity VAS suggests that accelerometry can still be used as a proxy for upper extremity activity. This correlation has not been described in the literature earlier. The significance of this correlation is, however, lost after correction for multiple comparisons. There are issues with correcting for multiple comparisons, with some statisticians recommending against this practice [55,56].

The excellent test-retest validity of WORC and QuickDASH has already been reported [12,42-45,52,57,58]. Our study adds new data on the excellent reliability of the SANE, pain VAS, and activity VAS in the context of shoulder pathologies. Similarly, the good intrajudge reliability of dynamometer-obtained shoulder strength and inclinometer-measured range of motion is confirmed in our study and resembles what has already been reported [13,59]. AT, AC, MIA, and HIA showed strong test-retest validity, which has not been reported in the literature in the context of shoulder pathology followed in an unrestricted home environment. Bruder et al [60] followed 15 distal radius fractures using wrist accelerometry and standardized tasks. Compared to our study, they obtained superior reliability for certain tasks such as classifying objects (ICC=0.83) and operating a lever (ICC=0.91), but similar or worse reliability for floor (ICC=0.69) and table cleaning (ICC=0.77) tasks and use of a keyboard (ICC=0.15). We hypothesize that the low reliability of LIA could be secondary to interference from other undesired movements detected by the accelerometers, but this remains to be tested.

As previously reported, WORC, QuickDASH, and pain VAS had excellent sensitivity to change and improvement in rotator cuff tendinopathy symptoms, both at 2 and 4 weeks after the intervention [46,52,53,61-66]. The SANE also showed excellent sensitivity to change, but only acceptable specificity to change. The activity VAS had moderate sensitivity to change at 2 and 4 weeks, and its low SRM on patients with low GRCS score indicated good specificity to change. Sensitivity to change of both SANE and activity VAS had not been previously reported in patients with shoulder pain or rotator cuff tendinopathy. As expected, sensitivity to change of clinical measures was low [15]. Sensitivity to change of all accelerometry measures was mediocre at 2 weeks, but acceptable at 4 weeks for AC, LIA, MIA, and HIA. This could be explained by a delay between the improvement in pain seen in questionnaires and patients increasing their use of the upper extremity. This delay could be secondary to previously acquired shoulder protective reflexes, slow improvement of a chronic condition, or no significant change in the patients’ daily routine following the intervention. Knowing this, wearing the accelometry device for longer period of time, for example, 6-8 weeks, might have shown better sensitivity, as patients may have increased their function over time. Sensitivity to change of wrist accelerometry in shoulder pathology has not been previously studied.

With a compliance of above 90%, participants seem to have had no issues in integrating the device in their daily routine. This adherence ratio is similar or superior than that reported in previous studies on the use of wrist accelerometers [35]. Very few complaints were voiced over the device and subjective acceptability seemed high. However, it is impossible to estimate how many potential participants declined the project because of the device. In a study on physical activity, 8.3% of participants refused to wear a wrist accelerometer for a duration of 9 days [67].

Data loss was larger than expected. Comparisons with other commercially available accelerometers show that these usually report between 3.3% and 10.8% of data loss [68-70]. Despite this setback, we were able to obtain enough data to calculate convergent validity, test-retest validity, and sensitivity to change of the wrist accelerometer. However, we recognize that this is an important limitation of the article, leading to possibly important bias in the data obtained, especially at the 4 weeks’ follow-up, where only 13 participants had enough combined data preinjection to allow analysis. The software malfunction has since been corrected for future studies, now yielding less than 1% data loss. Other improvements to the device could include better power management to increase recording time, miniaturize the device, and make it water resistant in order to increase comfort and adherence to accelerometer use in all activities. Low battery life of the device might have led to a bias where possibly important activity data at the end of the day was lost. Usability issues encountered in this embedded study with the activity and monitoring platform used have since been addressed by using smartwatches with motion sensors as a data logging platform for the proposed measurement approach. Possible uses of such a system could be the development of an app that allows clinicians and surgeons to follow the rehabilitation and progress of their patients in real-time, potentially allowing for less frequent clinical visits. The patient could also track his own progress to determine if more home physical therapy work is needed to remain in the correct recovery pathway. This could lead to decreasing health care cost for the patient, while allowing the clinician to free up more clinical time to see additional patients and decrease wait lists.

This study has multiple strengths. First, it is the first to report and compare metrological qualities of accelerometers in patients with shoulder pain or rotator cuff tendinopathy. Second, our strict inclusion criteria ensured internal validity of the study. Third, although we report similar data as those reported for the WORC and QuickDASH, we added significant information on pain and activity VAS, SANE, shoulder strength, range of motion, and accelerometry in the context of rotator cuff tendinopathy following a CSI. However, since we included only one pathology, the external validity of the study is diminished. The physical examination was performed by two different examiners, which might be a source of bias in this validation study.

### Conclusions

Daily pain VAS and SANE showed good correlation with validated questionnaires, excellent reliability at 1 week, and excellent sensitivity to change at 2 and 4 weeks. Daily activity VAS– and accelerometry-derived AT were well correlated. Activity VAS showed excellent reliability, but moderate sensitivity to change. Accelerometry measures have moderate reliability and moderate sensitivity to change at 4 weeks.

## Appendix

Multimedia Appendix 1 Daily questionnaire provided to participants, including a pain Visual Analog Scale, an activity Visual Analog Scale, and the Single Assessment Numeric Evaluation.

Multimedia Appendix 2 Accelerometer satisfaction questionnaire provided to participants.

## Abbreviations

AC: activity count
AT: active time
CHUS: Centre Hospital Universitaire de Sherbrooke (Sherbrooke University Health Center)
CSI: corticosteroid injection
GRCS: Global Rating of Change Score
HIA: high-intensity activity
LIA: low-intensity activity
MIA: medium-intensity activity
NSAID: nonsteroidal anti-inflammatory drug
PRO: patient-reported outcome
QuickDASH: short version of the Disability of the Arm, Shoulder, and Hand questionnaire
SANE: Single Assessment Numeric Evaluation
SRM: standardized response mean
tDCS: Transcranial Direct Current Stimulation
VAS: Visual Analog Scale
WIMU-GPS: Wireless Inertial Measurement Unit with GPS
WORC: Western Ontario Rotator Cuff index
