# Supplementary material for: Wrist-Based Accelerometers and Visual Analog Scales as Outcome Measures for Shoulder Activity During Daily Living in Patients With Rotator Cuff Tendinopathy: Instrument Validation Study
Source: JMIR Rehabil Assist Technol. 2019 Dec 3;6(2):e14468. doi: 10.2196/14468 (PMC6918212; doi:10.2196/14468)
Supplement: Multimedia Appendix 1 [file rehab_v6i2e14468_app1.pdf]

Service d'orthopédie
Fleurimont — Hôtel-Dieu

Daily questionnaire

Ne pas numériser. Si envoyé aux archives par mégarde, retourner au local 4109

Evaluation date : DD / MM / 20 _ _

Affected side : ❑ Right ❑ Left

Use : *To fill by patient*
For all upper limb pathology

Project Title: ______

IDENTIFICATION :

Patient Initials: ______

Patient Research Number: ______

Please rate your ability to do the following activites **in the last 24 hours**. *Please mark your answers with a slash "/"*

1. In the last 24 hours, how would you rate the pain or discomfort felt in your painful shoulder?

No pain |—|—|—|—|—|—|—|—|—|—| **Extreme** pain

2. In the last 24 hours, how much did you use your arm from which the shoulder is painful for your daily activities?

Not at all |—|—|—|—|—|—|—|—|—|—| **All the time**

3. How would you rate your shoulder today as a percentage of normal? (0 to 100% being normal)

__________ %

4. Please share any comments or difficulty with the use of the device :

____
____
____
____
