# Supplementary material for: Wrist-Based Accelerometers and Visual Analog Scales as Outcome Measures for Shoulder Activity During Daily Living in Patients With Rotator Cuff Tendinopathy: Instrument Validation Study
Source: JMIR Rehabil Assist Technol. 2019 Dec 3;6(2):e14468. doi: 10.2196/14468 (PMC6918212; doi:10.2196/14468)
Supplement: Multimedia Appendix 2 [file rehab_v6i2e14468_app2.pdf]

Service d'orthopédie
Fleurimont — Hôtel-Dieu

IMU questionnaire

Ne pas numériser. Si envoyé aux archives par mégarde, retourner au local 4109

Evaluation date : DD / MM / 20 _ _

Affected side : ❑ Right ❑ Left

Use : *To fill by patient*
For all upper limb pathology

Project Title: ______

IDENTIFICATION :

Patient Initials: ______

Patient Research Number: ______

Please answer the questions thinking about **the whole duration of the study**. *Please mark your answers with a slash " / "*

1. Thinking about the whole duration of the study, how would you rate the level of discomfort caused by the device worn at the wrist?

no discomfort |—|—|—|—|—|—|—|—|—|—| **extreme** discomfort

2. Thinking about the whole duration of the study, how would you rate the level of inconvenience caused by the act of wearing the device around your wrist?

no inconvenience |—|—|—|—|—|—|—|—|—|—| **extremely** inconvenient

3. How many days do you think you forgot to wear the device?

__________ day(s)

4. How many times do you think you forget to recharge the device at night?

__________ time(s)

5. Please share any comment you have about the device worn at the wrist:

____
____
____
____
